# Post-traumatic stress disorder after ICU discharge: results of a post-ICU aftercare program

**DOI:** 10.1186/cc14633

**Published:** 2015-03-16

**Authors:** D Ramnarain, I Gnirrip, W Schapendonk, A Rutten, G Van der Nat, N Van der Lely

**Affiliations:** 1 St.Elisabeth Hospital Tilburg, the Netherlands

## Introduction

Patients who survive ICU treatment may experience psychological distress for some time after discharge from the ICU. In the literature the reported prevalence of post-traumatic stress disorder (PTSD) ranges from 5 to 64% [1]. We studied PTSD symptoms in relation to ICU factors, demographic data and physical complaints reported by patients 4 to 6 months after ICU discharge.

## Methods

Patients who were treated in our ICU from 1 January 2013 until 31 December 2013 for more than 5 days were invited to visit our ICU aftercare clinic. Six weeks after discharge a letter of invitation together with a health-related questionnaire, the Hospital Anxiety and Depression Scale questionnaire and Impact of Event Scale (IES-R) questionnaire, was sent to the patient. Patients were asked to return the questionnaires prior to visiting the aftercare clinic. All data were analyzed and if the IES-R score indicated a possible PTSD, patients were referred to a psychologist for further analyses and treatment. All patient data were analyzed retrospectively. The Pearson chi-squared test was used to compare groups and Cramer's V analyses was used to examine strength of the association between groups.

## Results

Seventy-nine patients, 54 male and 43 women, with mean age 57 years. Median APACHE II and APACHE IV were 18 and 60 respectively. Median ICU days and hospital days were 9 and 20 respectively. Seventy-six percent of patients were mechanically ventilated with a median of 5 days. Median time of ICU discharge to aftercare visit was 165 days. Delirium occurred in 22 (22.7%) patients during ICU treatment. The prevalence of PTSD was 43.3% and was most seen in patients after subarachnoid hemorrhage (SAH) (28.6%). Pain, muscle weakness, fatigue, impairment in daily activity, dyspnea, and hoarseness reported during the ICU aftercare clinic visit were significantly associated with PTSD. There was no significant difference in men and women. Sedation, opiates, benzodiazepine, inotropic medication and delirium during ICU treatment were not associated with higher prevalence of PTSD. None of the other demographic data analyzed were significantly associated with PTSD.

## Conclusion

Prevalence of PTSD was 43.3% and most seen in patients after SAH, reflecting the majority of patients treated in our ICU. PTSD was associated significantly with pain, muscle weakness, fatigue, dyspnea, hoarseness and impairment of daily activity after a median 165 days post ICU treatment.

## References

[B1] Griffiths J Intensive Care Med 2007 33 1506 18 10.1007/s00134-007-0730-z 17558490

